# Anomalous Statistics of Bose-Einstein Condensate in an Interacting Gas: An Effect of the Trap’s Form and Boundary Conditions in the Thermodynamic Limit

**DOI:** 10.3390/e20030153

**Published:** 2018-02-27

**Authors:** Sergey Tarasov, Vladimir Kocharovsky, Vitaly Kocharovsky

**Affiliations:** 1 Institute of Applied Physics, Russian Academy of Science, Nizhny Novgorod 603950, Russia; 2 Department of the Advanced School of General and Applied Physics, Lobachevsky State University, Nizhny Novgorod 603950, Russia; 3 Department of Physics and Astronomy, Texas A&M University, College Station, TX 77843-4242, USA

**Keywords:** Bose-Einstein condensation, statistics of Bose-Einstein condensate, Bogoliubov coupling, interacting Bose gas, mesoscopic system

## Abstract

We analytically calculate the statistics of Bose-Einstein condensate (BEC) fluctuations in an interacting gas trapped in a three-dimensional cubic or rectangular box with the Dirichlet, fused or periodic boundary conditions within the mean-field Bogoliubov and Thomas-Fermi approximations. We study a mesoscopic system of a finite number of trapped particles and its thermodynamic limit. We find that the BEC fluctuations, first, are anomalously large and non-Gaussian and, second, depend on the trap’s form and boundary conditions. Remarkably, these effects persist with increasing interparticle interaction and even in the thermodynamic limit—only the mean BEC occupation, not BEC fluctuations, becomes independent on the trap’s form and boundary conditions.

## 1. Critical Phenomena and Statistics of Anomalous Fluctuations in a Bose-Einstein Phase Transition

Any second order phase transition in a many-body system occurs near its critical point via development of the critical phenomena and anomalously large fluctuations of an order parameter [1]. Finding their microscopic theory and analytical description constitutes one of the major and still unsolved problem in theoretical physics. A phase transition to the Bose-Einstein condensed state in the interacting Bose gas of *N* particles confined in an optical/magnetic trap is one of the phenomena that have been the most intensively studied in the last two decades (for a review, see [2] and references therein).

In the present paper we analyze the statistics of the number of condensed particles at the temperature *T* well outside a narrow critical region around the critical temperature $T_{c}$, namely, at $T\ll T_{c}$ where a well-known mean-field approximation based on the Gross-Pitaevskii and Bogoliubov-de Gennes equations is valid. (In fact, such a mean-field approximation works reasonably well even up to the temperature $T_{c}/2$, that is for all temperatures in the interval $T<T_{c}/2$, as has been verified, for example, by the experiments on the dynamics and decay of the condensate excitations [2,3,4,5,6].) At somewhat higher temperatures, but still outside the critical region, one could employ a more complicated theory (beyond the mean field) that consistently accounts for a deeper thermal depletion of spatially inhomogeneous condensate and various many-body effects of particle interactions via an approximate solution to the Dyson equation for the Green’s and self-energy functions of the standard quantum-field-theory method [3,4]. Even higher temperatures at the wings of critical region, which are still far from the central part of critical region, could be accessed by means of a phenomenological renormalization-group theory (for a review, see [7] and references therein). Finally, the BEC phase transition in the weakly interacting Bose gas at any temperatures outside and inside the entire critical region can be rigorously described by the general equations for the order parameter, self-energy and Green’s functions within a recently developed microscopic theory of phase transitions [8,9,10].

The study of the BEC statistics has a long and rich history (see, for example, an arbitrary sample of relatively recent papers [11,12,13,14,15,16,17,18,19,20,21,22,23,24,25] and references therein.) However, nobody succeeded yet in deriving the BEC statistics in the interacting gas for the temperature interval $T_{c}/2<T<T_{c}$ and the entire critical region neither by means of the standard quantum-field-theory method, renormalization-group theory or microscopic theory nor by means of the other known approaches (discussed, e.g., in [6]) which are more involved than the Bogoliubov approximation.

An analytical solution for the BEC statistics at any temperature, including the entire critical region, was found recently [15,26,27,28,29,30] only for the ideal, non-interacting gas confined in a trap of arbitrary geometry and dimensions. In particular, it provides a universal scaling of all thermodynamic quantities and a universal form of the BEC occupation probability distribution over the entire critical region in the thermodynamic limit of macroscopically large systems. This universal statistics is either non-Gaussian of an anomalously large variance or Gaussian, depending on the trap geometry, and clearly manifests itself in the mesoscopic systems starting from the number of particles *N* of the order of a thousand.

An essential novelty of the work [15] and the subsequent papers [26,27,28,29,30] was a formulation (and a solution) of the very problem of finding the entire probability distribution and its characteristic function for the BEC statistics as opposed to the calculation of just the first two statistical moments, the mean value and the variance, discussed in the preceding works (see, e.g., [11,13,14,18,23]). It was done mainly for the ideal Bose gas, with only one exception. Namely, in the paper [15] we put forward and solved this problem within the Bogoliubov approximation for a weakly interacting gas confined in a three-dimensional (3D) box trap with the periodic boundary conditions. Such a trap has a purely homogeneous ground state and a homogeneous condensate wave function.

In the present paper we continue this line of research. Namely, we find the probability distribution and the characteristic function, including all moments and cumulants, for the BEC statistics in a weakly interacting dilute gas confined in a 3D rectangular box of dimensions $L_{x},L_{y},L_{z}$ with the Dirichlet (zeroth) or so-called fused boundary conditions (see (28) and thereinafter). In the Dirichlet’s case a one-particle ground state is strongly inhomogeneous $\propto \sin\left(\pi x/L_{x}\right)\sin\left(\pi y/L_{y}\right)\sin\left(\pi z/L_{z}\right)$ and one has to solve the Gross-Pitaevskii and Bogoliubov-de Gennes equations for the inhomogeneous condensate and quasiparticle wave functions (see, e.g., [2,3,5,6,7,31,32]). For simplicity’s sake, we do this within the Thomas-Fermi approximation assuming that the healing length ξ is much less than the box dimensions:

(1) $$\xi \ll L_{x},L_{y},L_{z};\;\xi =\sqrt{\frac{V}{8\pi N_{0}a}}\;\;,\;g=\frac{4\pi \hbar^{2}a}{M}\;\;.$$

Here $N_{0}$ is the mean number of particles in the condensate, *M* the mass of a particle, $V=L_{x}L_{y}L_{z}$ the volume of a trap, a>0 the s-wave scattering length determining the interaction constant *g*. If the first-order Born approximation for the two-body repulsive interaction with a potential $U(r_{1}-r_{2})$ is applicable, one can think of the interaction constant as of the k=0 component, $g=U_{0}$, of the Fourier transform $U_{k}=\int U\left(r\right)\exp\left(ikr\right)d^{3}r$ of the two-body potential and model the latter by a Dirac delta-function $U\left(r\right)=U_{0}\delta \left(r\right)$. The diluteness of the gas means its low density $N/V\ll a^{-3}$, i.e., a small value of the gas parameter $Na^{3}/V\ll 1$. This assumption of the dilute gas together with the Thomas-Fermi condition (1) implies the following inequalities on the admissible values of the condensate gas parameter $n_{0}a^{3}$:

(2) $$\frac{1}{\sqrt{8\pi }}\frac{a}{L}\ll \sqrt{n_{0}a^{3}}\ll 1\;\mathrm{for}\;\;L=L_{x},L_{y},L_{z};\;n_{0}=\frac{N_{0}}{V}.$$

Then, possessing the BEC statistics for the mesoscopic system of *N* interacting particles in the box traps of different rectangular forms and different, periodic, fused or Dirichlet, boundary conditions, we compare their statistics and conclude on the effect of the trap’s form and boundary conditions on them. In particular, we calculate the asymptotics of the BEC statistics and the effect of the trap’s form and boundary conditions in the thermodynamic limit N→∞, V→∞ with a concentration of particles N/V being kept constant.

The paper is organized as following. In Section 2 we formulate the basic model of the BEC statistics in a weakly interacting gas confined in a flat trap within the mean-field theory of the Gross-Pitaevskii equation for the condensate and Bogoliubov—de Gennes equations for the quasiparticles, both being simplified by means of the Thomas-Fermi approximation. In Section 3 we present the general analytical formulas for the characteristic function and all cumulants of the noncondensate occupation probability distribution for a mesoscopic system of *N* particles in a weakly interacting gas in the case when the Bogoliubov coupling squeezes (forces to coherently correlate the particles’ creation and annihilation operators) the contribution to the noncondensate occupation separately either from each quasiparticle or from each pair of symmetric counter-propagating quasiparticles. In Section 4 we derive the explicit analytical formulas for the asymptotics of the obtained characteristic function and all cumulants of the noncondensate occupation probability distribution in the thermodynamic limit. In Section 5 we apply these results for BEC statistics to the case of the cubic or rectangular box trap with the periodic, fused or Dirichlet boundary conditions in the regime of thermally-dominated fluctuations. Section 6 contains the discussion and implications of the obtained results for the statistical physics of the BEC phase transition. The main conclusions are stated in Section 7.

## 2. Quasiparticles: The Spectrum and Eigenstates Within the Gross-Pitaevskii and Bogoliubov—De Gennes Equations in the Thomas-Fermi Approximation

In the thermal equilibrium the mesoscopic system of *N* interacting particles is described by the statistical operator (density matrix)

(3) $$\hat{\rho }=e^{-\hat{H}/T}\prod_{j}\left(1-e^{-\varepsilon_{j}/T}\right),\;\hat{H}=\sum_{j}\varepsilon_{j}{\hat{b}}_{j}^{†}{\hat{b}}_{j},$$

where the Hamiltonian $\hat{H}$ is given by the Bogoliubov approximation as the sum over the dressed by the condensate and many-body interaction quasiparticles with the eigenenergies $\varepsilon_{j}$ and creation (annihilation) operators ${\hat{b}}_{j}^{†}$ (${\hat{b}}_{j}$) enumerated by the index j=1,2,…. The field operator of the particles

(4) $$\hat{\psi }\left(r\right)=\sqrt{N_{0}}\phi \left(r\right)+\hat{\theta }\left(r\right),\;\mathrm{where}\;\hat{\theta }=\sum_{j}\;\left[u_{j}\left(r\right){\hat{b}}_{j}+v_{j}^{*}\left(r\right){\hat{b}}_{j}^{†}\;\right],\;\left[{\hat{b}}_{j},{\hat{b}}_{j^{\prime }}^{†}\right]=\delta_{j,j^{\prime }},$$

includes the c-number (classical) condensate wave function $\sqrt{N_{0}}\phi \left(r\right)$ and the field operator $\hat{\theta }\left(r\right)$ of the noncondensate particles. The operators ${\hat{b}}_{j}^{†}$ and ${\hat{b}}_{j}$ obey the canonical commutation relations.

The profile of the condensate wave function ϕ (which is chosen to be real) obeys the Gross-Pitaevskii equation [2]

(5) $$\left(-\frac{\hbar^{2}}{2M}\Delta_{r}+U_{trap}\left(r\right)+gN_{0}\phi^{2}\left(r\right)-\mu \right)\phi \left(r\right)=0,\;\int \phi^{2}\left(r\right)d^{3}r=1,$$

where $U_{trap}\left(r\right)$ is the external potential of the trap and μ the chemical potential. The eigenenergies and eigenstates ${\left(u_{j},v_{j}\right)}^{T}$ of quasiparticles are determined by the Bogoliubov-de Gennes equations [2,31,32]

(6) $$\left(-\frac{\hbar^{2}}{2M}\Delta_{r}+U_{trap}\left(r\right)+2gN_{0}\phi {\left(r\right)}^{2}-\mu \right)\left(\begin{array}{c} u_{j}\\ v_{j} \end{array}\right)+gN_{0}\phi^{2}\left(r\right)\left(\begin{array}{c} v_{j}\\ u_{j} \end{array}\right)=\varepsilon_{j}\left(\begin{array}{c} +u_{j}\\ -v_{j} \end{array}\right)$$

with the boundary conditions specified by the trap setup. Here $\Delta_{r}$ is the Laplace operator.

The operator of the number of noncondensed particles that determines the statistics of the condensate depletion can be represented via the creation and annihilation operators of quasiparticles:

(7) $${\hat{N}}_{ex}\equiv \int {\hat{\theta }}^{+}\hat{\theta }d^{3}r=\sum_{j,k}\left({\hat{b}}_{j}^{+}{\hat{b}}_{k}\int u_{j}^{*}u_{k}d^{3}r+{\hat{b}}_{j}{\hat{b}}_{k}\int u_{j}v_{k}d^{3}r+{\hat{b}}_{j}^{+}{\hat{b}}_{k}^{+}\int u_{j}^{*}v_{k}^{*}d^{3}r+{\hat{b}}_{j}{\hat{b}}_{k}^{+}\int v_{j}v_{k}^{*}d^{3}r\right).$$

The subscript “ex” stands for “excitations”. Since the total number of particles in the trap is conserved, N=const, the condensate occupation statistics is determined by the complimentary operator ${\hat{N}}_{0}=N-{\hat{N}}_{ex}$. The parameter $N_{0}$, that enters the Equations (5) and (6) and plays a part of the mean field in this Bogoliubov approach, is the mean condensate occupation and should be found from the self-consistency equation

(8) $$N_{0}=N-\langle {\hat{N}}_{ex}\rangle ,\;\langle \ldots \rangle =\mathrm{Tr}\left(\ldots \hat{\rho }\right).$$

The angle brackets denote averaging over the quantum ensemble given by the statistical operator (3).

In fact, the particle-number-conservation constraint ${\hat{N}}_{0}+{\hat{N}}_{ex}=N$ defines the canonical or microcanonical (along with the energy constraint $\hat{H}=E=const$) statistical ensembles and is responsible for the very existence of the BEC phase transition as a process leading to a macroscopic occupation of a single quantum state [1,2,9,31]. Thus, a rigorous microscopic description of the BEC requires an introduction of the constraint Hilbert space by an appropriate truncation of the original Fock space of the system of *N* particles. The most natural and straightforward way of doing so is to express all physical quantities via the particle-number-conserving creation and annihilation operators of the canonical quasiparticles which describe the transitions between the ground and excited one-particle states. Such operators were first introduced in the famous Holstein-Primakoff representation [33] (worked out also by Schwinger [34]) and are known in the theory of BEC as the Girardeau-Arnowitt operators [35,36]. The similar “number-conserving” approaches were widely discussed in the literature – for example, see [37] introducing the ladder operators similar to the Girardeau-Arnowitt ones (but conserving the particle number only approximately, [36]), [38] involving the modified ladder operators to establish the U(1)-symmetry model for calculating the dynamics of BEC, [39] formulating the number-conserving finite temperature theory which takes into account the non-quadratic terms of Hamiltonian, [15] describing the BEC statistics in the Bogoliubov approximation via the Girardeau-Arnowitt operators, [16,17] describing the higher-order cumulants of the BEC statistics in an ideal gas at zero temperature, [40,41] presenting the description of the coupled dynamics of the condensate and noncondensate fractions, [24] calculating the correlations in a Bose gas via introduction of the c-number constants for the Girardeau-Arnowitt operators and the so-called “coherent” energy levels which are highly-occupied and mostly form the condensate mode of the interacting gas, [19] where the number-conserving approach was used for a comparatative analysis of the generalized BEC in the anysotropic boxes within the canonical and grand-canonical ensembles, etc.

While the particle number conservation is crucially important for the correct description of BEC in the critical region [9,26,27,28,29], the constraint ${\hat{N}}_{0}+{\hat{N}}_{ex}=N$ is less important for the well-developed condensate phase at low temperatures $T\ll T_{c}$ considered in the present paper. Here the role of the constraint is reduced mainly to the self-consistency Equation (8) for the mean condensate occupation and has only a minor effect on fluctuations. In fact, the particle number conservation does not affect directly and cannot eliminate the strong effect of squeezing of quasiparticle contributions to the condensate depletion in Equation (7) via the Bogoliubov couplings.

The Bogoliubov-de Gennes system of equations is not a standard realization of the Sturm–Liouville problem, and there is no usual orthogonality of the partial *u*’s and *v*’s components of quasiparticle wave functions in the general case [31,32]. This fact is crucial for the existence of the nonzero Bogoliubov couplings, that is the nonzero integrals playing a part of coefficients in the right hand side of Equation (7). Actually, the sensitivity of the BEC statistics to the trap’s form and boundary conditions strongly involves the statistics’ dependence on the spectrum of quasiparticles, while the aforementioned non-orthogonality of the partial components of eigenfunctions $u_{j}$ and $v_{j}$ determines the squeezing of quasiparticle contributions to the condensate depletion in Equation (7) via the Bogoliubov couplings. At the same time, the eigenvectors ${\left(u_{j},v_{j}\right)}^{T}$ of the quasiparticle wave functions are orthogonal [31] in the sense of the difference-component scalar product:

$$\int \left(u_{i}u_{j}^{*}-v_{i}v_{j}^{*}\right)dV=\delta_{i,j},\;\int \left(u_{i}v_{j}-u_{j}v_{i}\right)dV=0,\;\int \left(u_{i}\phi -v_{i}\phi^{*}\right)dV=0.$$

Thus, finding the statistics of the condensate depletion operator ${\hat{N}}_{ex}$ over the quantum statistical ensemble $\hat{\rho }$ is a problem, far from being trivial, of diagonalizing an infinite matrix (7) with mostly nonzero entries defined by the overlapping integrals of quasiparticle states. However, there are cases when only a small part of the overlapping integrals is significant. One of such cases is the case of the flat traps discussed below.

In the present paper we simplify calculations by employing the Thomas-Fermi approximation in which one ignores the kinetic energy term $-\left(\hbar^{2}/\left(2M\right)\right)\Delta_{r}$ in the Gross-Pitaevskii Equation (5) and obtain the analytical solution for the condensate wave function:

(9) $$\sqrt{N_{0}}\phi \left(r\right)\approx \sqrt{\frac{\mu -U_{trap}\left(r\right)}{g}}\;\mathrm{if}\;U_{trap}\left(r\right)<\mu$$

and zero elsewhere. It is known that this approximation works quite well if the interparticle interaction is strong enough for the condition (1) to be fulfilled [2,32]. Moreover, we restrict analysis to the case of flat traps with a constant value (taken to be zero) of trapping potential inside the entire trap’s volume and the infinite potential walls at the trap’s borders. According to the Thomas-Fermi approximation (9), it amounts to the homogeneous profile of the condensate, $\phi \left(r\right)=V^{-1/2}$, and $\mu =gn_{0}$. In this case the solution to the Bogoliubov-de Gennes Equations (6) for the quasiparticle states acquires the form

(10) $$u_{j}\left(r\right)=\frac{f_{j}\left(r\right)}{\sqrt{1-A_{j}^{2}}},\;v_{j}\left(r\right)=\frac{A_{j}f_{j}\left(r\right)}{\sqrt{1-A_{j}^{2}}}$$

with the spatial profile of both components $u_{j}\left(r\right)$ and $v_{j}\left(r\right)$ given by the same function $f_{j}\left(r\right)$ which is the solution to the one-particle Shrödinger equation of the empty (without condensate) trap:

(11) $$-\frac{\hbar^{2}}{2M}\Delta_{r}f_{j}\left(r\right)=\epsilon_{j}^{\left(0\right)}f_{j}\left(r\right).$$

The Shrödinger Equation (11) establishes the orthogonal basis of the one-particle wave functions $\left\{f_{j}\left(r\right)\right\}$ and the corresponding spectrum of the bare energies: $\epsilon_{1}^{\left(0\right)}\leq \epsilon_{2}^{\left(0\right)}\leq \epsilon_{3}^{\left(0\right)}\leq \ldots$

We assume that the latter equation is equipped with the same boundary conditions as were imposed on the one-particle wave function at the borders of the original (without condensate) trap. This means that the quasiparticles are mostly determined by the Bogoliubov-de Gennes equations applied to the central part of the almost homogeneous condensate which demonstrates a significant density drop only in the relatively thin, of the order of the healing length $\xi \ll L_{x},L_{y},L_{z}$, inhomogeneous boundary layers. At the same time, the boundary conditions imposed at the borders of the trap are important and strongly influence the quasiparticles in the following ways. First, the high-energy quasiparticles, which have the quasi-classical (WKB) properties, propagate adiabatically (freely) through the boundary layers, that is, they carry a full information about the boundaries and fully “feel” the boundary conditions. Second, the low-energy quasiparticles, the wavelengths of which are of the order of the trap’s dimension, also know about boundaries since even in the central part of the volume these quasiparticles must obey the symmetry of the system which is determined by the boundary conditions. For example, switching between the Dirichlet and periodic boundary conditions, roughly speaking, enables or disables a half of all “wavevector components” (quantum numbers) in the spectrum of eigenstates in each spatial direction. Since the BEC statistics is determined mainly by the large groups of excited states and the trending terms of energy spectra, we assume that the corrections to eigenenergies $\epsilon_{j}^{\left(0\right)}$ caused by the inhomogenety of the condensate in the boundary layers via varying coefficients in the Bogoliubov-de Gennes Equations (6) do not strongly affect the BEC statistics in the asymptotics of vanishing parameter $\xi /V^{1/3}\to 0$. Thus, although the boundary condensate layers are formally omitted in the Thomas-Fermi approximate solution (9), they are actually present in the basic model formulated above. They are responsible for a transition from the homogeneous value of the condensate profile $\phi \left(r\right)=V^{-1/2}$ in the trap’s volume to the value (for example, zero in the Dirichlet’s case) required by the original boundary conditions at the trap’s borders and also determine the symmetry of the whole system.

The model (10) and (11), based on the Thomas-Fermi approximation, of the Bogoliubov-de Gennes Equations (6) describes the quasiparticles with the spectrum of eigenenergies $\varepsilon_{j}$ and Bogoliubov couplings determined by the coupling parameter $A_{j}$ given by the following equations:

(12) $$\varepsilon_{j}=\sqrt{{\left(\epsilon_{j}^{\left(0\right)}\right)}^{2}+2gn_{0}\epsilon_{j}^{\left(0\right)}},\;A_{j}=\frac{\varepsilon_{j}-\epsilon_{j}^{\left(0\right)}-gn_{0}}{gn_{0}},$$

where the interaction coupling energy $gn_{0}$ is proportional to the condensate density $n_{0}=N_{0}/V$. Similar approaches are known in the literature on the BEC in the inhomogeneous systems and were used by many authors (see, e.g., [32,42]) for the calculation of quantum depletion and other parameters of BEC.

It is worth noting that the formulated above model for the BEC statistics (10)–(12) is very basic and poses a series of further questions which go beyond the scope of the present paper. They include, for example,

(i) the problem of finding the actual spectrum and Bogoliubov couplings of quasiparticles from the solutions of the exact Gross-Pitaevskii and Bogoliubov-de Gennes Equations (5) and (6) beyond the Thomas-Fermi approximate model (10)–(12) as well as more specific questions, like
(ii) the problem of the possibly missed or excessive quasiparticles in the model (10)–(12),
(iii) the problem of an accurate transfer of the actual trap’s boundary conditions into the equivalent boundary conditions for the approximate Shrödinger Equation (11) for quasiparticles at the borders of the main body of the quasi-homogeneous condensate through the inhomogeneous condensate boundary layer of thickness $\;\xi \ll L_{x},L_{y},L_{z}$ (for example, in terms of the impedance boundary conditions dictated by the exact solution to the Gross-Pitaevskii Equation (5)).

A possible approach to these problems, based on the second-order perturbation theory, can be found in [42] where the Bogoliubov analysis of the weakly inhomogeneous Bose system as well as the results for the corresponding deformation of the condensate and for the expectation value of the quantum depletion were presented. In particular, a suppression of the quantum depletion induced by a weak lattice was predicted. (The effect is probably related to the quasiparticle selection and corresponding limitation of quasiparticle spectrum by means of the Bragg scattering on the lattice.)

We’ll discuss these problems and effects elsewhere. Below we focus on the technique for the analysis of BEC statistics in the interacting gas within the basic model (10)–(12) and, in particular, on a possible effect of the trap’s form and boundary conditions on the statistics of condensate depletion in the thermodynamic limit.

## 3. The Characteristic Function and Cumulants of the BEC Depletion Probability Distribution for a Mesoscopic System of Weakly Interacting Particles

Here we describe a technique for evaluating the statistics of the total number of noncondensed particles. The statistics is derived in terms of a Fourier transform of its characteristic function which is described via the cumulants and generating cumulants related to the BEC depletion operator ${\hat{N}}_{ex}$ in Equation (7) and calculated analytically.

We consider a mesoscopic system with any finite number *N* of trapped particles and dimensions of the trap $L_{x},L_{y},L_{z}$. Let us introduce dimensionless energy variables:

(13) $$\begin{array}{c} \lambda_{j}^{\left(0\right)}\equiv \epsilon_{j}^{\left(0\right)}/\epsilon_{1}^{\left(0\right)},\;\lambda_{j}\equiv \varepsilon_{j}/\epsilon_{1}^{\left(0\right)},\;\Delta \equiv gn_{0}/\epsilon_{1}^{\left(0\right)},\;\alpha \equiv \epsilon_{1}^{\left(0\right)}/T,\\ \mathrm{so}\;\mathrm{that}\;\;\epsilon_{j}^{\left(0\right)}/T=\alpha \lambda_{j}^{\left(0\right)},\;\varepsilon_{j}/T=\alpha \lambda_{j},\;gn_{0}/T=\alpha \Delta . \end{array}$$

The dimensionless spectra $\left\{\lambda_{j}^{\left(0\right)}\right\}$ and $\left\{\lambda_{j}\right\}$ represent the bare one-particle and renormalized by condensate quasiparticle energies, respectively. The strength of the interparticle interaction is characterized by the parameter $\Delta =gn_{0}/\epsilon_{1}^{\left(0\right)}$ which is the ratio of the interaction coupling energy to the energy gap in the bare one-particle spectrum. The applicability condition of the Thomas-Fermi approximation (1) means that one has Δ≫1. The dimensionless parameter α characterizes the size of the system, so that the thermodynamic limit of a macroscopically large system means α→0. The statistical operator (3) and Bogoliubov quasiparticle eigenenergies $\varepsilon_{j}$ and coupling coefficients $A_{j}$ of Equation (12) acquire the following forms with an explicit appearance of the asymptotic parameter α:

(14) $$\hat{\rho }=e^{-\alpha \sum_{j}\lambda_{j}{\hat{b}}_{j}^{+}{\hat{b}}_{j}}\prod_{j}\left(1-e^{-\alpha \lambda_{j}}\right),\;\lambda_{j}=\sqrt{{\left(\lambda_{j}^{\left(0\right)}\right)}^{2}+2\lambda_{j}^{\left(0\right)}\Delta },\;A_{j}=\frac{\lambda_{j}-\lambda_{j}^{\left(0\right)}-\Delta }{\Delta }.$$

The present analysis implies the Bogoliubov approximation which is valid at $T\ll T_{c}$ (or maybe even up to $T<T_{c}/2$), that is, anyway far outside the critical region. Hence, the condensate is well developed and the particle number constraint of the canonical ensemble $N={\hat{N}}_{0}+{\hat{N}}_{ex}$ does not play any significant role for the occupation numbers of the quasiparticle states and different blocks of quasiparticles (which are, in fact, pairs of quasiparticles in the case of the box trap), which have mutually nonzero overlapping integrals in the excited particle occupation operator (7), produce independent contributions to the noncondensate occupation. This means that the characteristic function is the product of the partial characteristic functions, corresponding to the occupation contributions from such blocks of quasiparticles, and the probability distribution ρ(n) of the condensate depletion operator ${\hat{N}}_{ex}$ could be effectively obtained via the Fourier transform of this characteristic function

(15) $$\Theta \left(u\right)\equiv \langle e^{iu{\hat{N}}_{ex}}\rangle ,\;\rho \left(n\right)=\frac{1}{2\pi }\int_{-\pi }^{\pi }e^{-iun}\Theta \left(u\right)du.$$

Thus, it remains to calculate the partial characteristic function for the noncondensate occupation from each separate block of quasiparticles contributing to the noncondensate with mutually coherent correlations, or squeezing, due to Bogoliubov couplings in Equations (7) and (10). This is straightforward to do either by the algebraic method suggested in [15] or by the developed later, equivalent and more general method of the Wigner transform [43]. The result can be written as the product of contributions from each quasiparticle *j* as follows

(16) $$\Theta \left(u\right)=\prod_{j}\sqrt{\frac{z(A_{j})-1}{z\left(A_{j}\right)-e^{iu}}\times \frac{z(-A_{j})-1}{z\left(-A_{j}\right)-e^{iu}}},\;z\left(A_{j}\right)=\frac{A_{j}-e^{\alpha \lambda_{j}}}{A_{j}e^{\alpha \lambda_{j}}-1}.$$

The corresponding generating cumulants (which are defined via the following derivatives of the logarithm of the characteristic function) have a symmetric transparent form:

(17) $${\tilde{\kappa }}_{m}\equiv {\left.\frac{\partial^{m}\ln\Theta \left(u\right)}{\partial {\left(e^{iu}-1\right)}^{m}}\right|}_{u=0}=\frac{\Gamma (m)}{2}\sum_{j}\left[\frac{1}{{\left(z\left(A_{j}\right)-1\right)}^{m}}+\frac{1}{{\left(z\left(-A_{j}\right)-1\right)}^{m}}\right],$$

Γ(m) denotes the Euler gamma functions. Here the product and the sum run over all quasiparticles of the considered basic model (10)–(12). These formulas hold for any homogeneously condensed mesoscopic system, including both the case of the real-valued eigenfunction $f_{j}$ (when Bogoliubov coupling squeezes the contribution to noncondensate occupation from each quasiparticle separately and the corresponding matrix blocks in the noncondensate occupation operator (7) are 2×2 blocks) and the case of the complex-valued eigenfunction $f_{j}$ (when Bogoliubov coupling squeezes the contribution to noncondensate occupation from each pair of symmetric counter-propagating quasiparticles and the corresponding matrix blocks in the noncondensate occupation operator (7) are 4×4 blocks) as well as even the case when both real- and complex-valued eigenfunctions are simultaneously present among the quasiparticles.

The presented generating cumulants are very useful for the analysis of the BEC statistics. The ordinary cumulants $\kappa_{m}\equiv d^{m}\ln\Theta \left(u\right)/d{\left(iu\right)}^{m}{|}_{u=0}$ and the initial moments $\alpha_{m}\equiv \langle {\hat{N}}_{ex}^{m}\rangle$ immediately follow from the generating cumulants (for the other general relations see, e.g., [15,26,44]):

$$\kappa_{r}=\sum_{m=1}^{r}\sigma_{r}^{\left(m\right)}{\tilde{\kappa }}_{m},\;\alpha_{m}=\sum_{r=1}^{m}\sum^{\left(m,r\right)}{\left(m;a_{1},\ldots ,a_{m}\right)}^{\prime }\kappa_{1}^{a_{1}}\ldots \kappa_{m}^{a_{m}}.$$

Here $\sigma_{r}^{\left(m\right)}$ are the Stirling numbers of the second kind [44], ${\left(m;a_{1},\ldots ,a_{m}\right)}^{\prime }=m!/\left[{\left(1!\right)}^{a_{1}}a_{1}!\ldots {\left(m!\right)}^{a_{m}}a_{m}!\right]$ is a multinomial coefficient, and the sum $\sum^{\left(m,r\right)}$ runs over the non-negative integers $a_{1},\ldots ,a_{r}$ which satisfy the following two conditions: $a_{1}+2a_{2}+\ldots +ra_{r}=r$ and $a_{1}+\ldots +a_{r}=m$. In particular, the first four cumulants constitute themselves the four most important characteristics of the shape of the probability density function – the mean value, the variance (square of the standard deviation), the asymmetry and the excess:

(18) $$\begin{array}{c} \mathrm{Mean}=\langle {\hat{N}}_{ex}\rangle =\kappa_{1}={\tilde{\kappa }}_{1};\\ \mathrm{Variance}=\sigma^{2}=\kappa_{2}={\tilde{\kappa }}_{2}+{\tilde{\kappa }}_{1};\\ \mathrm{Asymmetry}=\kappa_{3}={\tilde{\kappa }}_{3}+3{\tilde{\kappa }}_{2}+{\tilde{\kappa }}_{1};\\ \mathrm{Excess}=\kappa_{4}+3\kappa_{2}^{2},\;\kappa_{4}={\tilde{\kappa }}_{4}+6{\tilde{\kappa }}_{3}+7{\tilde{\kappa }}_{2}+{\tilde{\kappa }}_{1}. \end{array}$$

The meaning and the main advantage of the cumulants are in their direct and explicit characterization of the non-Gaussian properties of statistics as well as its asymptotics at the wings of the probability density function. In particular, the cumulants of the Gaussian statistics are exactly zero for all orders higher than the second order. Thus, the simple analytical expressions (17) for the generating cumulants yield the analytical results for any other statistical parameters which one could be interesting in.

The Equation (17) for the first cumulant, m=1, has a special meaning. It is a nonlinear self-consistency Equation (8) for determining the BEC order parameter that is the BEC mean occupation:

(19) $$N_{0}=N-\sum_{j}\frac{1+A_{j}^{2}}{1-A_{j}^{2}}\frac{1}{e^{\alpha \lambda_{j}}-1}-\sum_{j}\frac{A_{j}^{2}}{1-A_{j}^{2}}.$$

This is because the total number of particles in the trap *N* is fixed, so that the occupation of condensate is determined by the number of excited particles, $N_{0}=N-\langle {\hat{N}}_{ex}\rangle$, and, at the same time, the mean number of excited particles $\langle {\hat{N}}_{ex}\rangle$ is determined via the Bogoliubov-de Gennes quasiparticles’ spectrum $\lambda_{j}$ and couplings $A_{j}$ which are governed by the interaction coupling energy $gN_{0}/V$. Formally, the self-consistency equation has a nontrivial solution not only in the whole region of validity of the Bogoliubov approximation at low enough temperatures, say, $T\ll T_{c}$, but also at the temperatures much closer to the critical temperature $T_{c}$ (see, for example, [15]). The solution to the self-consistency equation could be found as a power series in the asymptotic parameter α via the Mellin transform technique discussed, for example, in [27,28].

When the self-consistent value of the condensate occupation $N_{0}$ has been found, the Equations (16) and (17) analytically yields the BEC statistics of the mesoscopic system. This statistics is non-Gaussian as is clearly seen from the formulas for the higher-order cumulants which establish nonzero values.

## 4. Asymptotics of the BEC Statistics in the Thermodynamic Limit

Let us now calculate the thermodynamic limit of the BEC statistics (15) and (16) obtained above for the basic model (10)–(12). The crucial fact is that this BEC statistics, in general, is non-Gaussian and depends on the trap’s form and boundary conditions not only for a mesoscopic system, but even for a macroscopically large system in the thermodynamic limit.

The cumulant analysis provides the most convenient and transparent description of the BEC statistics. First of all, let us find the asymptotic scaling of the cumulants in the limit α≪1,Δ≫1 via a direct evaluation of the sum in the Equation (17). The two terms in the summand forming the generating cumulant ${\tilde{\kappa }}_{m}$ in the Equation (17) could be written in the following forms:

(20) $$\begin{array}{c} \frac{1}{z(+A_{j})-1}=\frac{1}{\lambda_{j}^{\left(0\right)}}\times \frac{\lambda_{j}}{e^{\alpha \lambda_{j}}-1}-\frac{A_{j}}{1+A_{j}},\\ \frac{1}{z(-A_{j})-1}=\frac{1}{\lambda_{j}^{\left(0\right)}+2\Delta }\times \frac{\lambda_{j}}{e^{\alpha \lambda_{j}}-1}+\frac{A_{j}}{1-A_{j}}. \end{array}$$

They are the combinations of the two summands which have a different origin and different dependencies on the two large parameters, $\alpha^{-1}\gg 1$ and Δ≫1.

The first terms in the right hand sides describe the effects of a nonzero temperature on the BEC statistics associated with the thermal depletion of BEC and strongly depend on the parameter α. They predictably vanish in the limit of very low temperatures T→0, or $\alpha \lambda_{1}\gg 1$, but play a major role in the moderate temperature regime. It is important to note that these “thermal” terms curiously involve the bare particle energies $\lambda_{j}^{\left(0\right)}$, which happens due to the exact algebraic relations $\left(1+A_{j}\right)/\left(1-A_{j}\right)=\lambda_{j}^{\left(0\right)}/\lambda_{j}$ and $\left(1-A_{j}\right)/\left(1+A_{j}\right)=\left(\lambda_{j}^{\left(0\right)}+2\Delta \right)/\lambda_{j}$ valid for the Bogoliubov spectrum (12) and its dimensionless counterpart (14).

The second terms in the right hand sides are responsible for the effects associated with the quantum depletion. They are totally independent on α (and temperature) and depend only on the interaction parameter Δ which determines the Bogoliubov couplings $A_{j}$.

Taking into account the asymptotic behavior of the coupling coefficient $A_{j}$,

(21) $$A_{j}≃-1+\sqrt{2\lambda_{j}^{\left(0\right)}/\Delta }\;\mathrm{for}\;\lambda_{j}^{\left(0\right)}\ll \Delta ,\;\mathrm{and}\;A_{j}≃-\Delta /2\lambda_{j}^{\left(0\right)}\;\mathrm{for}\;\;\lambda_{j}^{\left(0\right)}\gg \Delta ,$$

we immediately conclude that the term ${\left(z(+A_{j})-1\right)}^{-1}$ in Equation (17) determines the main contributions from the states with low energies, $\lambda_{j}^{\left(0\right)}\ll \Delta$, since it has the resonant denominators both in the “quantum” and “thermal” parts. For the states with high energies, $\lambda_{j}^{\left(0\right)}\gg \Delta$, both terms in brackets in Equation (17) are equally important.

For the first generating cumulant these “thermal” and “quantum” summands are present as the separate contributions which is clearly illustrated by Equation (19). However, for all higher-order generating cumulants there is an interplay of these “thermal” and “quantum” contributions in accord with the following binomial expansion:

(22) $${\tilde{\kappa }}_{m}=\frac{\Gamma (m)}{2}\sum_{j}\sum_{l=0}^{m}C_{m}^{l}\left(\frac{{\left(-A_{j}\right)}^{m-l}}{{\left(\lambda_{j}^{\left(0\right)}\right)}^{l}{\left(1+A_{j}\right)}^{m-l}}+\frac{{\left(A_{j}\right)}^{m-l}}{{\left(\lambda_{j}^{\left(0\right)}+2\Delta \right)}^{l}{\left(1-A_{j}\right)}^{m-l}}\right){\left(\frac{\lambda_{j}}{e^{\alpha \lambda_{j}}-1}\right)}^{l},$$

where $C_{m}^{l}$ stands for the binomial coefficient.

Let us consider separately the three significantly different cases corresponding to the three possible relations between the two large parameters, $\alpha^{-1}\gg 1$ and Δ≫1. The case of a very large interaction energy and low temperatures, $gn_{0}\gg T,\epsilon_{1}^{\left(0\right)}$, corresponds to a very cold, somewhat degenerate interacting gas with the negligible thermal effects. The opposite case of relatively high temperatures, $T\gg gn_{0}\gg \epsilon_{1}^{\left(0\right)}$, corresponds to an interacting gas with thermally dominated fluctuations. In the intermediate case the interplay between the thermal and quantum effects should be taken into account. The following analysis of the cumulants’ scaling requires a comparison of the contributions from various individual states (quasiparticles) to the noncondensate occupation and is closely related to the applicability of the central limit theorem to the corresponding random variable (7) which is the sum of many random squeezed contributions from the quasiparticles.

In the case of very low temperatures, the probability distribution of the noncondensate occupation is determined solely by the quantum effects, and all Bose thermal exponents in Equation (20) are negligibly small. The cumulants are scaled in terms of the large parameter Δ≫1. The individual contribution of a low-energy quasiparticle (with an almost linear, or acoustic, Bogoliubov-de Gennes spectrum $\lambda_{j}$) to the generating cumulant ${\tilde{\kappa }}_{m}$ has the order of ${\left(\Delta /\lambda_{j}^{\left(0\right)}\right)}^{m/2}$ which easily follows from the Equations (17) and (20). The bare energy $\lambda_{j}^{\left(0\right)}$ has a quadric dependence on the quantum numbers. Thus, the first two cumulants, $\langle {\hat{N}}_{ex}\rangle$ and $\sigma^{2}$, can be approximated by the well-convergent integrals over all states {j} that yields the scaling $\langle {\hat{N}}_{ex}\rangle ∼\sigma^{2}∼\Delta^{3/2}$. The corresponding sums are much larger than the individual contributions since a plenty of states contribute considerably. The higher-order cumulants cannot be approximated in the same way since the corresponding integrals are divergent at low energies. It means that only a small fraction of low-energy states makes the dominant contribution to the value of higher-order cumulants which has the same order of magnitude as the individual summands. In a result, the higher-order cumulants scale as $\kappa_{m}∼\Delta^{m/2}$ for m≥4. The third cumulant has a little bit different scaling $\kappa_{3}∼\Delta^{3/2}\ln\Delta$ which corresponds to the logarithmic divergence of the approximating integral and means that a number of high-energy states make a small, but not totally negligible contributions.

These calculations show that in the thermodynamic limit of the macroscopically large system, V→∞ at a constant condensate density, $N_{0}/V≃\mathrm{const}$, the total noncondensate occupation in a quantum-dominated regime experiences the standard thermodynamic fluctuations with the standard deviation $\sigma ∼\sqrt{\langle {\hat{N}}_{ex}\rangle }$ and has the normal, Gaussian probability distribution with vanishing normalized higher-order cumulants: $\kappa_{m}/\sigma^{m}\to 0$. Exactly the same situation holds for the statistics of the noncondensate occupation in an ideal Bose gas trapped in the three-dimensional harmonic potential [27,28] when the largest parameter in the system is $\alpha^{-1}$.

In the opposite case of thermally-dominated fluctuations, the thermal depletion is much larger than the quantum depletion. In this case the main contribution to the generating cumulants immediately follows from the binomial expansion (17) if one keeps only the largest terms:

(23) $${\tilde{\kappa }}_{m}=\frac{\Gamma (m)}{2}\sum_{j}\left[\frac{1}{{\left(\lambda_{j}^{\left(0\right)}\right)}^{m}}+\frac{1}{{\left(\lambda_{j}^{\left(0\right)}+2\Delta \right)}^{m}}\right]{\left(\frac{\lambda_{j}}{e^{\alpha \lambda_{j}}-1}\right)}^{m}.$$

(Let us stress again that the brackets in this expression involve the bare energy spectrum.) The individual “thermal” contribution of a quasiparticle to the generating cumulant ${\tilde{\kappa }}_{m}$ has the order of ${\left(\alpha \lambda_{j}^{\left(0\right)}\right)}^{-m}$ and demonstrates the dependence on $\lambda_{j}^{\left(0\right)}$ that is significantly steeper than the one revealed for the “quantum” contribution. Thus, for any 3D flat trap the sum could be obtained via the integral over all states {j} only for the first cumulant ${\tilde{\kappa }}_{1}$, which immediately yields the following result

(24) $$\langle {\hat{N}}_{ex}\rangle ≃V\;\zeta \left(\frac{3}{2}\right){\left(\frac{MT}{2\pi \hbar^{2}}\right)}^{3/2}∼\alpha^{-3/2},$$

where $\zeta \left(j\right)=\sum_{l=1}^{\infty }l^{-j}$ is a Riemann zeta function. It is worth noting that the contribution of each quasiparticle *j* is on the order of $\alpha^{-1}$, but a huge number of the contributing states makes the collective result much larger, namely, $\langle {\hat{N}}_{ex}\rangle ∼\alpha^{-3/2}$.

At the same time, for all higher-order cumulants including the second one (which gives the variance of the distribution) the corresponding integral is divergent at low energies. It means, that the main contribution to the value of ${\tilde{\kappa }}_{m}$ for m≥2 comes from a small enough number of the lowest energy quasiparticles *j*, and the discrete structure of the energy spectrum should be taken into account. Employing the series expansion for the Boltzmann exponent $e^{\alpha \lambda_{j}}-1≃\alpha \lambda_{j}$, we obtain

(25) $${\tilde{\kappa }}_{m}=\frac{\Gamma \left(m\right)\alpha^{-m}}{2}\sum_{j}\left[\frac{1}{\lambda_{j}^{(0)m}}+\frac{1}{{\left(\lambda_{j}^{\left(0\right)}+2\Delta \right)}^{m}}\right].$$

The Equation (25) means that the total value of the sum is of the same order as the values of the individual contributions. The latter result is similar to the one obtained in [27,28] for the ideal Bose gas. The only difference is that now, for the interacting gas, it involves the two spectral sums, $\sum_{j}\frac{\Gamma (t)}{\lambda_{j}^{(0)t}}$ and $\sum_{j}\frac{\Gamma (t)}{{\left(\lambda_{j}^{\left(0\right)}+2\Delta \right)}^{t}}$, instead of a single spectral sum $\sum_{j}\frac{\Gamma (t)}{{\left(\lambda_{j}-\lambda_{0}\right)}^{t}}$ that was employed in the BEC statistics of the ideal gas with the ground state energy $\lambda_{0}$. The Equation (25) for m=2 immediately reveals the scaling of the variance, $\sigma^{2}∼{\tilde{\kappa }}_{2}∼\alpha^{-2}$. Thus, the system is characterized by the anomalously large variance [11,12,14,15,18,25], $\sigma^{2}∼{\langle {\hat{N}}_{ex}\rangle }^{4/3}\gg \langle {\hat{N}}_{ex}\rangle$, as is clear from the Equations (24) and (25).

This, partly heuristic method of finding the cumulants’ asymptotics could be verified by a more rigorous method involving the Mellin transform and employed previously for the ideal gas in [27,28]. The latter method yields even the smaller next order terms of asymptotics omitted in (25). However, here we skip discussion of these terms and other details of the asymptotics. Let us just note that the generating cumulant ${\tilde{\kappa }}_{m}$ and the ordinary cumulant $\kappa_{m}$ have the same main term in the asymptotics.

It is convenient to introduce the normalized random variable $x=(N_{ex}-\langle {\hat{N}}_{ex}\rangle )/\sigma$ which characterizes the statistics of the number of noncondensed particles, has the zero mean value and the unity variance. The cumulants and characteristic function of this normalized variable could be easily obtained by applying the scaling and employing the trap function S(t,u) discussed in detail in [27]. The crucial point of the calculation is that the scaling of the variance $\sigma^{2}∼\alpha^{-2}$ is related to the scaling of the higher-order cumulants $\kappa_{m}∼\alpha^{-m}$ in quite a special way. Thus, in the thermodynamic limit α→0 the simple calculations of the scaled higher-order cumulants $\kappa_{m}^{\left(x\right)}$ of the normalized variable *x* yield the following explicit analytical result:

(26) $$\begin{array}{c} \kappa_{1}^{\left(x\right)}=0;\;\kappa_{2}^{\left(x\right)}=1;\;\kappa_{m}^{\left(x\right)}\equiv \frac{\kappa_{m}}{\sigma^{m}}=\frac{\tilde{S}\left(m\right)}{\tilde{S}{\left(2\right)}^{m/2}}\;\mathrm{for}\;m>2,\\ \tilde{S}\left(t\right)=\frac{\Gamma (t)}{2}\sum_{j}\left[\frac{1}{{\left(\lambda_{j}^{\left(0\right)}\right)}^{t}}+\frac{1}{{\left(\lambda_{j}^{\left(0\right)}+2\Delta \right)}^{t}}\right],\;\tilde{S}\left(t,u\right)=\frac{\Gamma (t)}{2}\sum_{j}\left[\frac{1}{{\left(\lambda_{j}^{\left(0\right)}-iu\right)}^{t}}+\frac{1}{{\left(\lambda_{j}^{\left(0\right)}+2\Delta -iu\right)}^{t}}\right], \end{array}$$

where we employed the trap function $\tilde{S}\left(t\right)$ and the extended trap function $\tilde{S}\left(t,u\right)$ determined by the spectrum $\left\{\lambda_{j}\right\}$. It is immediate to conclude that in the thermodynamic limit α→0 the scaled higher-order cumulants tend to the nonzero constants determined by the trap’s form (geometry) and boundary conditions as well as by the strength of the interparticle interaction.

The probability density function $\rho_{x}$ of the normalized random variable $x=(N_{ex}-\langle {\hat{N}}_{ex}\rangle )/\sigma$ can be written straightforwardly in the form of the Fourier transform:

(27) $$\rho_{x}=\frac{1}{2\pi }\int_{-\infty }^{\infty }e^{-iux}\Theta^{\left(x\right)}\left(u\right)du,\;\Theta^{\left(x\right)}\left(u\right)=\tilde{S}\left(0,\frac{u}{\sqrt{\tilde{S}\left(2\right)}}\right)-\tilde{S}\left(0\right)-\frac{iu}{\sqrt{\tilde{S}\left(2\right)}}\tilde{S}\left(1\right).$$

The characteristic function $\Theta^{\left(x\right)}\left(u\right)$ in Equation (27) is a well-defined function due to an exact cancellation of all singular terms (see the Appendix in [27]) and appears to be non-Gaussian. Thus, within the basic model (10)–(12), the probability distribution ρ(n) of the noncondensate occupation (15) and, hence, the probability distribution of the complimentary condensate occupation are significantly non-Gaussian for any number *N* of interacting particles and any dimensions of the trap, and the difference with the Gaussian statistics is significant.

In the intermediate case, both thermal and quantum contributions to the cumulants, which are fused in the binomial formula (22), are important and should be taken into account. The Gaussian or non-Gaussian behavior of the BEC statistics is closely related to the way the second cumulant, or the variance, $\kappa_{2}\equiv \sigma^{2}$, is accumulated. Its “thermal” contribution scales as $\alpha^{-2}$, and its “quantum” contribution scales as $\Delta^{3/2}$. They are of the same order, that is $\sigma ∼\alpha^{-1}∼\Delta^{3/4}$, when $\alpha \Delta^{3/4}∼1$. At the same condition, all higher-order cumulants $\kappa_{m}$, m≥3, are mostly determined by the thermal contribution which is of the order of $\alpha^{-m}$. (The quantum contributions are of the same importance only for $\alpha \Delta^{1/2}∼1$ which corresponds to a stronger interparticle interaction.) Hence, the scaled higher-order cumulants $\kappa_{m}^{\left(x\right)}$ tend in the thermodynamic limit to some nonzero constants which implies the non-Gaussian statistics. However, the difference from the Gaussian statistics is somewhat suppressed compared to the thermally-dominated regime due to a larger value of the variance. This non-Gaussian distribution is characterized by the standard deviation $\sigma ∼\sqrt{\langle {\hat{N}}_{ex}\rangle }$ which is not anomalously large yet. It happens so because the first cumulant, $\kappa_{1}=\langle {\hat{N}}_{ex}\rangle$, is mostly determined by the quantum contribution. The thermal contribution to $\langle {\hat{N}}_{ex}\rangle$ becomes of the same order as the quantum one only for αΔ∼1 which corresponds to the higher temperatures.

In summary, the non-Gaussian statistics of the total noncondensate occupation clearly appears in the 3D flat traps starting from $\alpha \Delta^{3/4}∼1$ and remains such for all smaller values of α, that is, in other words, starting from the temperature $T∼\sqrt[{4}]{{\left(gn_{0}\right)}^{3}\epsilon_{1}^{\left(0\right)}}$ and for the higher temperatures. The quantum-dominated and thermally-dominated regimes correspond to the large and small values of the same dimensionless parameter, $\alpha \Delta^{3/4}\gg 1$ and $\alpha \Delta^{3/4}\ll 1$, respectively.

The same conclusions are true for the large enough mesoscopic systems (that is, when α is small, but finite) since the relative corrections to the leading asymptotic terms discussed above become much less than 1 when the thermodynamic-limit parameter α and the inverse particle-interaction parameter $\Delta^{-1}$ become much less than unity: $\alpha \ll 1,\;\Delta^{-1}\ll 1$. Thus, the scaled statistics of the BEC in the mesoscopic systems is quite similar to the thermodynamic-limit statistics.

## 5. Statistics of the BEC Depletion in a Weakly Interacting Gas Trapped in the Cubic or Rectangular Box with the Periodic, Dirichlet or Fused Boundary Conditions in the Regime of Thermally-Dominated Fluctuations

The most curious and nontrivial regime among the ones described above is the regime of thermally-dominated fluctuations where the central limit theorem is not applicable and the BEC statistics is non-Gaussian. In the present section we consider this regime closely and present the detailed analysis of the corresponding probability density function in the thermodynamic limit. For simplicity’s sake, we employ mostly graphics and skip the long analytical calculations. For the mesoscopic, but relatively large systems (that is, when α≪1, say, $\alpha ∼10^{-2}$) the scaled BEC statistics is close enough to the one in the thermodynamic limit and could be easily obtained, including all finite-size corrections, on the basis of the mesoscopic values of cumulants given by Equations (17).

We start with some general properties of the probability density function $\rho_{n}$ or its scaled counterpart $\rho_{x}$, (27), described by the basic model (10)–(12), common for all three-dimensional cubic or rectangular box traps, no matter what are the boundary conditions. The leading term of the asymptotics of the characteristic function at the large values of the argument is given by a power law $\Theta_{x}\left(u\right)∼{\left(-iu\right)}^{3/2}$ as could be shown in the same way as it was done in [27,28] for the case of an ideal gas. It means that the actual distribution $\rho_{x}$ is described by an exponential slope $\rho_{x}∼e^{-x}$ for the positive arguments *x*, i.e., for $n>\langle {\hat{N}}_{ex}\rangle$, and by much steeper, super-exponential slope $\rho_{x}∼e^{-{\left|x\right|}^{3}}$ for the negative arguments *x*, i.e., for $n<\langle {\hat{N}}_{ex}\rangle$. The form of both wings of the probability distribution is quite different from the Gaussian law.

The origin of the strongly non-Gaussian statistics is closely related to an inapplicability of the central limit theorem. The point is that the statistical distribution and the cumulants of the noncondensate occupation are dominated by the anomalously large contributing occupations (random variables) of a relatively small number of the lowest energy quasiparticles. The inapplicability of the central limit theorem also means that the shape of the probability distribution could be affected by a modification of a relatively small fraction of the quasiparticle energy spectrum, for example, caused by some perturbation of the trap’s form or boundary conditions.

Let us illustrate this general conclusion by a straightforward comparison of the probability distributions of the total noncondensate occupation for three, in general, anisotropic box traps of dimensions $L_{x}\leq L_{y}\leq L_{z}$ with the periodic, Dirichlet or fused boundary conditions, respectively. According to the basic model (10)–(12), these traps possess the bare spectra $\alpha \lambda_{j}^{\left(0\right)}\equiv \epsilon_{j}^{\left(0\right)}/T$ enumerated by the integer vector $j=(j_{x},j_{y},j_{z})$, namely,

(28) $$\begin{array}{c} \alpha^{\left(p\right)}=\frac{2\hbar^{2}\pi^{2}}{ML_{z}^{2}T}\;,\;\lambda_{j}^{\left(0)(p\right)}=\frac{L_{z}^{2}}{L_{x}^{2}}j_{x}^{2}+\frac{L_{z}^{2}}{L_{y}^{2}}j_{y}^{2}+j_{z}^{2},\;j_{x,y,z}=\pm 0,\pm 1,\pm 2,\ldots ,\;\mathrm{excluding}\;j=\left(0,0,0\right)\\ \alpha^{\left(D\right)}=\frac{\hbar^{2}\pi^{2}}{2ML_{z}^{2}T}\;,\;\lambda_{j}^{\left(0)(D\right)}=\frac{L_{z}^{2}}{L_{x}^{2}}j_{x}^{2}+\frac{L_{z}^{2}}{L_{y}^{2}}j_{y}^{2}+j_{z}^{2},\;j_{x,y,z}=+1,+2,+3,\ldots \;,\\ \alpha^{\left(f\right)}=\frac{\hbar^{2}\pi^{2}}{2ML_{z}^{2}T}\;,\;\lambda_{j}^{\left(0)(f\right)}=\frac{L_{z}^{2}}{L_{x}^{2}}j_{x}^{2}+\frac{L_{z}^{2}}{L_{y}^{2}}j_{y}^{2}+j_{z}^{2},\;j_{x,y,z}=0,+1,+2,\ldots ,\;\;\mathrm{excluding}\;j=\left(0,0,0\right). \end{array}$$

Here and hereafter we denote the quantities specific to the periodic, Dirichlet or fused boundary conditions by the superscripts (p), (D), or (f), respectively. Note that the parameters $\alpha^{\left(p\right)}$ for the box with the periodic boundary conditions is 4 times larger than the Dirichlet’s or fused trap’s $\alpha^{\left(D\right)}$ and $\alpha^{\left(f\right)}$ because for this box the lowest-energy nontrivial one-particle state should demonstrate a full wavelength spatial variance along the trap, while the Dirichlet (or any impedance) boundary conditions allow also a half wavelength one-particle states. The periodic boundary conditions are introduced mainly for the convenience of theoretical calculations. In the actual experiments, usually the Dirichlet boundary conditions are more relevant. Since the true effective boundary conditions for the Shrödinger Equation (11) are not fully understood yet, we model them not only by the classical Dirichlet (zero) boundary conditions which prohibit the zero quantum values for any of the quantum numbers $j_{x},j_{y},j_{z}$, but also by the so-called fused boundary conditions which allow for the zero quantum numbers and combine some but not all features of the periodic and Dirichlet boundary conditions. Including such zero quantum numbers in the quasiparticle energy spectrum has a direct physical meaning as allowing for the entire planes, like $\left(0,j_{z},i_{z}\right)$, or lines, like $\left(0,0,j_{z}\right)$, of quasiparticles more closely related to the state j=(0,0,0) corresponding to the trivial, zero-energy Goldstone mode u(r)=−v(r)=ϕ(r). This mode corresponds to an arbitrary phase of the condensate wave function and, therefore, does not contribute to the noncondensate occupation. However, it is known that the Goldstone mode should always be present among the solutions of the Bogoliubov-de Gennes equations [18,32,45]. This fact suggests that the quasiparticles with the entire lines or planes of the zero quantum numbers (see Equation (28)) could also be present in the system. Note also that the most significant difference between the three spectra in Equation (28) is the difference in the ranges (28) in which the quantum numbers $\left(j_{x},j_{y},j_{z}\right)$ vary.

First, let us elaborate, by means of the analytical result in Equations (27) and (28), on the values of the cumulants and their dependence on the interaction strength parameter Δ (see Equation (13)) for the case of the cubic box traps, $L_{x}=L_{y}=L_{z}=L$.

The box with the periodic boundary conditions provides 8 times larger number of quasiparticles compared to the Dirichlet’s one because the integer vectors $j=(j_{x},j_{y},j_{z})$ enumerating the quasiparticles for the box with periodic boundary conditions fill all 8 octants of the three-dimensional integer lattice compared to the only one positive octant filled by the integer vectors enumerating the quasiparticles for the Dirichlet’s box. However, since the energies (and, hence, the parameter $\alpha^{\left(p\right)}$) for the periodic boundary conditions are 4 times larger and the leading term in the asymptotics of the first cumulant $\kappa_{1}∼\alpha^{-3/2}$ scales as the 3/2 power of $\alpha^{-1}$, the thermodynamic limit of the mean noncondensate occupation is the same, no matter which boundary conditions are applied.

The eightfold shortage of the number of quasiparticles is not the only important thing which happens as one alters the boundary conditions. Another significant fact is that the change of the boundary conditions disproportionately adds or removes all quasiparticles with the zero quantum numbers. That group of quasiparticles is quite large since in the sphere of quantum numbers $\left(j_{x},j_{y},j_{z}\right)$ with energies no larger than Λ, $\lambda_{j}\leq \Lambda$, there are $∼\Lambda^{2/3}$ quasiparticles with the zero quantum numbers. This observation explains a pronounced effect of the boundary conditions on all higher-order cumulants which appear to be noticably different for the different traps. Numerically, the variance and asymmetry in a cubic trap, $L_{x}:L_{y}:L_{z}=1:1:1$, are as follows

(29) $$\begin{array}{c} \kappa_{2}^{\left(p\right)}:\kappa_{2}^{\left(D\right)}:\kappa_{2}^{\left(f\right)}=\frac{{\tilde{S}}_{2}^{\left(p\right)}}{16}:{\tilde{S}}_{2}^{\left(D\right)}:{\tilde{S}}_{2}^{\left(f\right)}=0.57:0.35:2.62,\\ \kappa_{3}^{\left(p\right)}:\kappa_{3}^{\left(D\right)}:\kappa_{3}^{\left(f\right)}=\frac{{\tilde{S}}_{3}^{\left(p\right)}}{64}:{\tilde{S}}_{3}^{\left(D\right)}:{\tilde{S}}_{3}^{\left(f\right)}=0.13:0.066:3.56, \end{array}$$

where the calculations were done for the interaction strength $\Delta^{\left(D,f\right)}\equiv gn_{0}/\epsilon_{1}^{\left(0)(D,f\right)}\equiv 2MgN_{0}/\left(\hbar^{2}\pi^{2}L\right)=80$ in the case of the boxes with the Dirichlet or fused boundary conditions (see Equation (28)) that corresponds to $\Delta^{\left(p\right)}\equiv gn_{0}/\epsilon_{1}^{\left(0)(p\right)}\equiv MgN_{0}/\left(2\hbar^{2}\pi^{2}L\right)=20$ in the case of the box with the periodic boundary conditions.

The values of the cumulants significantly depend on the parameter Δ which characterizes the strength of interparticle interaction. However, even in the limit of very strong interaction Δ→∞ (i.e., $gn_{0}\gg \epsilon_{1}^{\left(0\right)}$) the higher-order cumulants keep nonzero values, so that the probability distribution remains non-Gaussian. The point is that with increasing Δ the contribution of a spectral sum with the shifted energies $\lambda_{j}+2\Delta$ in Equation (26) decreases. As a result, the values of “the amplitudes” $\tilde{S}\left(m\right)$ which determine the asymptotic values of the cumulants $\kappa_{m}$ in the thermodynamic limit tend to a half of their values at Δ=0. (Note that the zero value of the interaction strength parameter, Δ=0, corresponds to a limit of the ideal gas that is admissible within the basic model (10)–(12) only for the trap with the periodic boundary conditions where the condensate is strictly homogeneous. The moderate or small values of Δ∼1 or ≪1 have only a formal meaning for the traps with the other boundary conditions since for the inhomogeneous condensate the Thomas-Fermi approximation (1) requires Δ≫1.) This decrease of the higher-order cumulants $\kappa_{m}$ can be described analytically via the following asymptotics of their “amplitudes” (the universal numbers)

(30) $$\tilde{S}\left(m\right)\to \frac{1}{2}\left[\tilde{S}\left(m\right){|}_{\Delta =0}+\frac{k\;\pi^{3/2}L_{x}L_{y}}{L_{z}^{2}}\times \frac{\Gamma \left(m-3/2\right)}{{\left(2\Delta \right)}^{m-3/2}}\right]\;\mathrm{at}\;\Delta \gg 1$$

with the coefficient *k* equal to 1/8 or 1 for the Dirichlet or periodic boundary conditions, respectively. We see that “the amplitudes” $\tilde{S}\left(m\right)$ of the cumulants of the orders m=3,4,5,… decrease by almost two times and reach their ultimate minimum values already at the moderate interaction strength, $MgN_{0}/\left(2\hbar^{2}\pi^{2}L\right)∼5$, which corresponds to Δ∼5 and Δ∼20 for periodic and Dirichlet boundary conditions, respectively.

**Figure 1:**
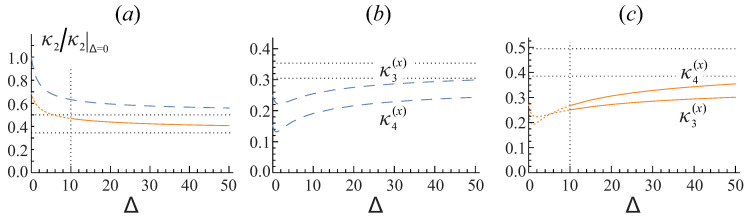
The dependences of cumulants on the interaction strength $\Delta \equiv gn_{0}/\epsilon_{1}^{\left(0\right)}$ in the thermodynamic limit of a very large system (α→0): (**a**) the variance $\kappa_{2}$ for a cubic trap with the periodic (a dashed blue line) and Dirichlet (a solid orange line) boundary conditions normalized to its (hypothetical) value $\kappa_{2}{|}_{\Delta =0}$ at zero interaction strength; (**b**) the normalized cumulants $\kappa_{3}^{\left(x\right)}$ and $\kappa_{4}^{\left(x\right)}$ for a cubic trap with the periodic boundary conditions and their asymptotic values at Δ→∞; (**c**) the normalized cumulants $\kappa_{3}^{\left(x\right)}$ and $\kappa_{4}^{\left(x\right)}$ for a cubic trap with the Dirichlet boundary conditions and their asymptotic values at Δ→∞. The curves for the Dirichlet’s trap are dotted in the region Δ<10 where the Thomas-Fermi approximation is expected to be inaccurate.

Importantly, the situation is opposite for the variance $\kappa_{2}$ and the corresponding value of its “amplitude” $\tilde{S}\left(2\right)$ which decreases very slowly, namely, as $1/\sqrt{2\Delta }$. This is demonstrated in Figure 1a: Even at Δ=50 the variance does not quite reach its ultimate minimum value. Note that for the case of the periodic boundary conditions at small Δ the variance tends exactly to the value known for the ideal Bose gas as it should be since in this case the condensate is homogeneous for any strength of repulsive interaction and, hence, the basic model is valid for any values of Δ. The situation is different for the case of the Dirichlet boundary conditions where the formal value of $\kappa_{2}$ at Δ=0 is 30% less than the true value of the ideal-gas variance.

The evolution of the normalized asymptotic cumulants $\kappa_{m}^{\left(x\right)}$ shown in Figure 1b,c in accord with the analytical results (27) and (28) clearly follows from the evolution of their “amplitudes”—the universal numbers $\tilde{S}\left(m\right)$. Namely, for small enough values of Δ (which has a physical meaning only for the case of the periodic boundary conditions) both $\tilde{S}\left(m\right)$ and $\tilde{S}\left(2\right)$ decrease with an increase of Δ. However, the larger the exponent in the spectral sum, the faster it goes down. As a result, the normalized cumulant $\kappa_{m}^{\left(x\right)}$, which is equal to the ratio $\tilde{S}\left(m\right)/\tilde{S}{\left(2\right)}^{m/2}$, decreases. For the moderate values of Δ the values of $\tilde{S}\left(m\right)$ at m=3,4,5… are already halved, but the value of $\tilde{S}\left(2\right)$ still is decreasing slowly. Therefore, the normalized cumulant slowly grows up to the level of $2^{m/2-1}\kappa_{m}^{\left(x\right)}{|}_{\Delta =0}$. In fact, that means that even for a large value Δ∼ 100–500 one should not totally neglect the second shifted spectral sum of the terms containing $\lambda_{j}+2\Delta$ in Equation (26). However, we’ll see below that the governed by these cumulants dependence of the probability distribution function on the value of the interaction strength parameter Δ is not very fast and is driven almost entirely by the Δ-dependence of the variance.

**Figure 2:**
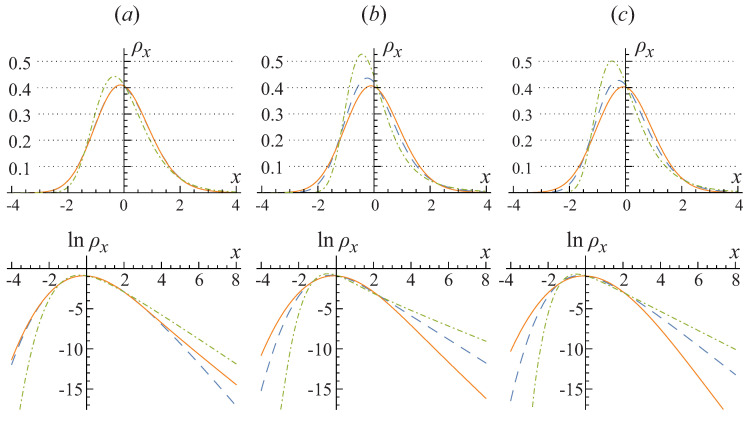
The normalized asymptotic distribution $\rho_{x}$ of the scaled total number of noncondensed particles, $x=(n-\langle {\hat{N}}_{ex}\rangle )/\sigma$, plotted in the thermodynamic limit (α→0) for different rectangular traps in the linear (up) and log (down) scales. The aspect ratio of a trap $L_{x}:L_{y}:L_{z}$ is (**a**) 1:1:1; (**b**) 1:1:2; and (**c**) 0.33:1:1. The dashed blue, solid orange and dot-dashed green curves are for the periodic, Dirichlet and fused boundary conditions, respectively. The interaction strength is $\Delta^{\left(D,f\right)}\equiv 2MgN_{0}/\left(\hbar^{2}\pi^{2}L\right)=80$ in the case of the boxes with the Dirichlet or fused boundary conditions (see (28)) that corresponds to $\Delta^{\left(p\right)}\equiv MgN_{0}/\left(2\hbar^{2}\pi^{2}L\right)=20$ in the case of the box with the periodic boundary conditions.

The result in Equation (30) demonstrates that the variance and asymmetry calculated for the traps with different boundary conditions are not the same. Therefore, in the general case the scaled asymptotic distributions for the corresponding traps do not coincide as well. It is also worth to note that changing the boundary conditions is not the only way to affect the higher-order cumulants. For example, the squeezing of a cubic trap to a prolonged or shortened rectangular box results into changing the asymptotic cumulants and significantly affects the scaled probability distributions. Both of these effects are illustrated in Figure 2. More general deformation of the trap geometry, for example, into some kind of a cylindrical form also would alter the noncondensate occupation statistics.

An effect of the trap’s form (geometry) on the probability distribution of the noncondensate occupation can be explicitly shown via dependence of the asymptotics of the characteristic function $\Theta^{\left(x\right)}\left(u\right)$ at the large values of its arguments, |u|≫1, on the dimensions $L_{x},L_{y},L_{z}$ of the anisotropic box trap. Indeed, we find that this asymptotics

(31) $$\begin{array}{cc} \ln\Theta^{\left(x\right)}\left(u\right) & ∼F\left(\frac{-iu}{\sqrt{\tilde{S}\left(2\right)}}\right)+F\left(\frac{-iu}{\sqrt{\tilde{S}\left(2\right)}}+2\Delta \right)\;,\;\mathrm{where}\\ F(y) & =\frac{2\sqrt{\pi }R_{3/2}\;y^{3/2}}{3}+\frac{R_{1}\;y\ln y}{2}+\frac{y}{2}\left({\tilde{S}}^{\left(0\right)}\left(1\right)-R_{1}+\gamma R_{1}\right)+\ldots \;, \end{array}$$

depends on the ratios $L_{x}:L_{y}:L_{z}$ via the *R*’s dimensionless coefficients given by the following formulas

(32) $$\begin{array}{cc} R_{3/2}^{\left(p\right)} & =\frac{\pi^{3/2}L_{x}L_{y}}{L_{z}^{2}},\;R_{1}^{\left(p\right)}=0,\\ R_{3/2}^{\left(D\right)} & =\frac{\pi^{3/2}L_{x}L_{y}}{8\;L_{z}^{2}},\;R_{1}^{\left(D\right)}=-\frac{\pi }{8}\left(\frac{L_{x}L_{y}}{L_{z}^{2}}+\frac{L_{x}}{L_{z}}+\frac{L_{y}}{L_{z}}\right),\\ R_{3/2}^{\left(f\right)} & =\frac{\pi^{3/2}L_{x}L_{y}}{8\;L_{z}^{2}},\;R_{1}^{\left(f\right)}=+\frac{\pi }{8}\left(\frac{L_{x}L_{y}}{L_{z}^{2}}+\frac{L_{x}}{L_{z}}+\frac{L_{y}}{L_{z}}\right), \end{array}$$

for the periodic, Dirichlet and fused boundary conditions, respectively (see Equation (28)). The coefficient ${\tilde{S}}^{\left(0\right)}\left(1\right)$ entering function F(y) in Equation (31) is a regularized (if it is singular) value of the trap function $\tilde{S}\left(t\right)$ at t=1, and γ≃0.577 is the Euler–Mascheroni constant.

**Figure 3:**
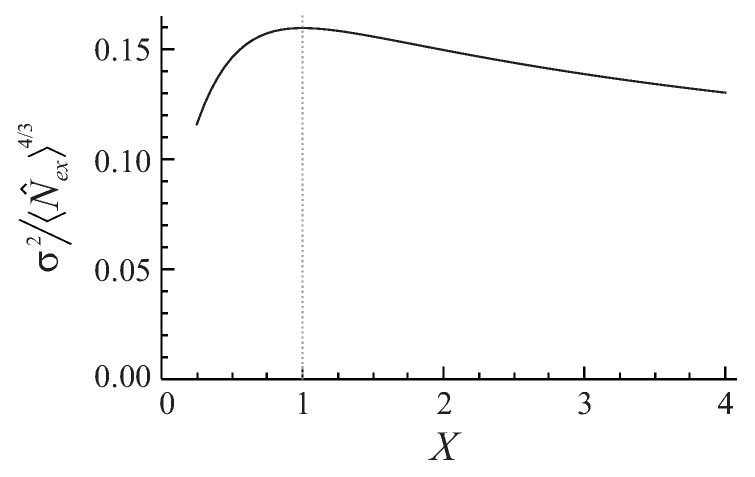
The thermodynamic-limit (α→0) asymptotics of the anomalously large variance $\sigma^{2}$, Equation (33), of the condensate depletion fluctuations in the interacting gas trapped in the rectangular box with the Dirichlet boundary conditions vs the box trap anisotropy characterized by the dimensions’ ratios $L_{x}:L_{y}:L_{z}=1:1:X$, where 0.25<X<4. The interaction strength is set to be $\Delta^{\left(D\right)}\equiv gn_{0}/\epsilon_{1}^{\left(0)(D\right)}=80$.

The change of the rectangular box form via the dimensions’ ratios $L_{z}:L_{y}:L_{z}$ affects “the variance amplitude” ${\tilde{S}}_{2}$ which is the characteristic scale of the variable *u*. (This is also clearly seen from the expression for the $\Theta^{\left(x\right)}\left(u\right)$ in Equation (27)). A simple analysis of the Fourier integral (27) shows that the quantity ${\left({\tilde{S}}_{2}\right)}^{-1/2}$ is the characteristic scale of the argument *x* of the scaled asymptotic distribution $\rho_{x}$. The dimensions’ ratios also determine the coefficients in the asymptotics (31) of $\Theta^{\left(x\right)}\left(u\right)$ at the large values of the argument. All these coefficients, starting from the next-to-leading term coefficient $R_{1}$, strongly depend on the dimensions’ ratios. Only the coefficient in front of the leading term could not be changed by means of the anisotropic squeezing or stretching of the box form if the volume of the trap is kept constant. (One has to take into account that the eightfold difference in Equation (32) between the coefficient $R_{3/2}^{\left(p\right)}$ and the coefficients $R_{3/2}^{\left(D\right)}$, $R_{3/2}^{\left(f\right)}$ for the periodic and the other two boundary conditions is exactly compensated by the difference in the definitions of $\alpha^{\left(p\right)}$ and $\alpha^{\left(D\right)}$, $\alpha^{\left(f\right)}$, see Equation (28)). The increasing anisotropy of the trap significantly decreases “the amplitude” ${\tilde{S}}_{2}$ of the anomalously large variance

(33) $$\sigma^{2}≃\frac{{\tilde{S}}_{2}}{{\left(R_{3/2}\right)}^{4/3}\;\zeta^{4/3}\left(3/2\right)}{\langle {\hat{N}}_{ex}\rangle }^{4/3}$$

since the leading term in the asymptotics of the mean value $\langle {\hat{N}}_{ex}\rangle$ does not feel the anisotropy and remains almost constant. Figure 3 demonstrates this effect for a very large, thermodynamic-limit rectangular box trap with the Dirichlet boundary conditions: the variance decreases by 20% when any dimension of the box becomes about four times longer (or shorter) than the other dimensions.

The result in the Equation (33) explicitly proves that the fluctuations of the condensate depletion in the weakly interacting gas are anomalously large compared to the standard thermodynamic fluctuations. The thermodynamic fluctuations would have considerably smaller variance, $\sigma^{2}∼\langle {\hat{N}}_{ex}\rangle$, proportional only to the first power of the mean value, while the variance (33) of the true noncondensate occupation is proportional to the larger power, $\sigma^{2}∼{\langle {\hat{N}}_{ex}\rangle }^{4/3}$, of the mean noncondensate occupation $\langle {\hat{N}}_{ex}\rangle \gg 1$. Thus, the basic model (10)–(12) is in agreement with this known fact [14,15,18].

The main effect of the change of boundary conditions is that a large group of states with one zero quantum number appears to be added or removed disproportionately. As a result, the next-to-leading logarithmic term appears in the characteristic function (31) that leads to the logarithmic shift of an argument in the exponent, which could make the left-wing decay of the distribution considerably faster or slower. However, for the cases considered in Figure 2 this effect is not a major one since in these cases the variance values ${\tilde{S}}_{2}$ differ so significantly (which, in fact, is not the general case!) that the most important effect of the trap’s form on the noncondensate occupation probability distribution consists in altering the characteristic scale of the noncondensate occupation random variable *x*.

The change of the boundary conditions also considerably affects the lowest energy quasiparticles which determine the right-wing asymptotics of the noncondensate occupation probability distribution. The smaller is the lowest energy and the higher is the corresponding degeneracy, the weaker exponential decay is realized for the larger than average noncondensate occupations x>0 (that is, for $n>\langle {\hat{N}}_{ex}\rangle$).

It is worth noting that a relatively pronounced effect of the change of the boundary conditions in the rectangular trap from the periodic to Dirichlet ones calculated above cannot be directly observed in the experiments since the periodic boundary conditions constitute an auxiliary theoretical model. Only some partial analogues of such switching of boundary conditions could be realized in a real system, for example, in a toroidal trap with an azimuthal symmetry and, hence, the periodic boundary condition along the azimuthal direction via cutting the torus by a high, almost vertical potential wall. This or similar cylindrically symmetric setups which are more relevant to the actual experiments will be discussed elsewhere. In the present paper we just draw attention to such effect of boundary conditions on the BEC statistics.

The overall picture of the effects of the trap’s form and boundary conditions on the noncondensate occupation statistics for a weakly interacting gas within the basic model (10)–(12) is similar to the one known for an ideal gas. (For example, statistics of BEC in an ideal gas trapped in the anisotropic box or slabs was discussed in [19,21,22,27,46].) As is illustrated in Figure 2, a typical change in the thermodynamic-limit asymptotics of the noncondensate occupation probability distribution due to a change of the trap’s form or boundary conditions is about 10% or so. Note that this is already normalized and centered probability distribution, so that the change cannot be eliminated by some kind of mesoscopic effects or perturbations.

Of course, the leading terms in the thermodynamic-limit asymptotics for the mean value $\langle {\hat{N}}_{ex}\rangle$ are the same for the different trap’s forms and boundary conditions. This happens since a very large number of the quasiparticles contribute to $\langle {\hat{N}}_{ex}\rangle$ and the individual contributions are not really important. The non-Gaussian distribution appears to be a robust property of the system—the trends of asymptotics could not be changed without changing the index in the power law for the density of states, and modification of a small number of quasiparticles hardly affects the behavior of the probability distribution. The effect of boundary conditions on the distributions is mostly governed by the large enough group of energies with one zero quantum number. The effect of the trap’s form deformation disproportionately affects the whole energy spectrum of quasiparticles and, hence, is significant. The same mechanisms determine the BEC statistics in an ideal gas as is shown in [27,28].

A further analysis beyond the basic model (10)–(12) could reveal the other cases when a change of the trap affects the BEC statistics significantly. For example, the effect of a change of the trapping potential in the entire volume of the trap, say, by means of imposing an additional weak potential lattice considered in [42], at moderate (but still less than critical, $T\ll T_{c}$) temperatures could be analyzed by the perturbation method of [42]. Unfortunately, the authors of [42] discussed only the case of the quantum-dominated regime at very close to zero temperatures when the BEC statistics is Gaussian in the bulk limit. Moreover, the ref. [42] is devoted to the calculation of the mean value (i.e., only the first cumulant) of quantum depletion, while we are interesting in the entire statistics (i.e., all higher-order cumulants) of condensate depletion and, mostly, in the opposite case of the thermally-dominated fluctuations when the statistics is significantly non-Gaussian even for the macroscopically large system. Note, however, that the dependence of the low-temperature limit of the mean condensate depletion on the large interaction parameter Δ, calculated in [42], is definitely in agreement with the “quantum” contribution to the first cumulant calculated in the present paper from Equation (25).It is possible, in principle, to calculate the characteristic function of the BEC statistics starting from Equations (29) and (31) of [42]. We expect that in the result one will obtain the regimes of the quantum-dominated, Gaussian and thermally-dominated, non-Gaussian fluctuations similar to the ones described in the present paper. Thus, these calculations could reveal the dependence of BEC statistics on the lattice perturbation of the trapping potential and provide a basis for the design of an actual experiment on its measurement.

## 6. Discussion: Why the BEC Statistics Remains Non-Gaussian and Dependent on the Trap’s Form and Boundary Conditions Even in the Thermodynamic Limit and Upon Increasing Interaction?

The proposed basic model (10)–(12) of the BEC statistics is relatively simple. It takes into account the main physical mechanism of an influence of the interparticle interaction on the noncondensate (and complimentary condensate) occupation and its fluctuations. The point is that the noncondensate occupation is a random variable equal to the sum of many bare-particle excited-state occupations created by the independently fluctuating Bogoliubov-de Gennes quasiparticles, as is clearly seen from the Equations (16) and (17). The interparticle interaction, mediated by the condensate, forces, via the Bogoliubov coupling, each quasiparticle or pair of counter-propagating quasiparticles to populate the bare-particle excited states in a squeezed, or coherently correlated mode. This is exactly what the basic model describes via the Equations (7) and (10).

Thus, on the one hand the basic model admits the nontrivial analysis presented in the Section 3, Section 4 and Section 5 and, in particular, the analytical solution for its thermodynamic-limit asymptotics. On the other hand, it allows one to take into account the effect of the interparticle interaction in the Bose gas and, in particular, the renormalization (dressing) of the quasiparticles and trap by a well-developed condensate fraction. As a result, this basic model yields a series of interesting conclusions about the probability distribution, moments and cumulants of BEC fluctuations as the functions of the interaction strength, temperature, the number of trapped particles, the dimension of the system, the form (geometry) of the trap and the imposed boundary conditions.

First of all, the noncondensate occupation fluctuations in an interacting gas are anomalously large compared to the standard thermodynamic fluctuations as is proven by the result in the Equation (33).

The second, but very important fact is that the BEC statistics turns out to be strongly non-Gaussian, as it was in the case of the non-interacting, ideal gas [15,26,27,28,29,30], and remains so with an increase of the interparticle interaction. The origin of this non-Gaussianness is, of course, in an inapplicability of the central limit theorem. Indeed, if the contribution of quasiparticles to the noncondensate occupation decreases slowly with an increase of their quantum numbers $j_{x},j_{y},j_{z}$, a large number of quasiparticles contributes significantly to the statistics, the central limit theorem works and one finally obtains the Gaussian distribution. In the opposite case of rapidly decreasing contributions the group of quasiparticles which mainly determines the statistics is not large enough and the central limit theorem is not applicable. Hence, the resulting statistics is non-Gaussian. This is exactly the picture behind the analysis of the values of cumulants and the characteristic function as the sums of the accumulating contributions from the lattice of quasiparticle states presented in the Section 3, Section 4 and Section 5.

As is known for the case of an ideal Bose gas [27,28], the three-dimensional systems with the quadratic spectrum, $\lambda_{j}∼j_{x}^{2}+j_{y}^{2}+j_{z}^{2}$, and the linear spectrum, $\lambda_{j}∼j_{x}+j_{y}+j_{z}$, constitute the examples of the “rapidly growing” and “slowly growing” spectra, respectively. The crucial nontrivial fact about the BEC statistics in a weakly interacting gas is as follows. The Bogoliubov-de Gennes quasiparticles have a “slowly growing” spectrum (12), $\varepsilon_{j}$, of an almost linear dependence of eigenenergies on the quantum numbers vector j starting from the zero energy until the interaction coupling energy $gn_{0}$. Nevertheless, the statistics of the noncondensate occupation (consisting of the excited bare particle occupations) is accumulated (due to the squeezing caused by Bogoliubov coupling) in accord with the bare energies $\epsilon_{j}^{\left(0\right)}$ (see the first sum in the square brackets of Equation (25)). But the bare energies form the quadratic spectrum and, hence, grow fast enough to disable the central limit theorem!

Next, we find that in the region of parameters where the thermal depletion is much larger than the quantum depletion the noncondensate occupation probability distribution converges in the thermodynamic limit to the special function determined by the trap’s form and boundary conditions. Within the basic model, the effect of the trap’s form and boundary conditions on the probability distribution could exist even in the thermodynamic limit. Moreover, any change of the spectrum, which strongly affects (or even excludes, as it happens when the boundary conditions are changed from the periodic to Dirichlet ones) a large enough group of quasiparticles, results into the probability distribution altering. This is the reason why the system geometry and dimensionality are very important for the BEC statistics. In particular, a proper choice of the trap’s geometry and a change of dimensionality from D=3 value to the lower, quasi-1D or quasi-2D values, for which the non-Gaussian BEC statistics is known [27] to be more pronounced and achievable starting from the lower exponent p=2D/(4−D) of the power-law trapping potential $U_{trap}∼{\left|r\right|}^{p}$, can increase the observable effect on the condensate depletion statistics.

The other mechanism of the influence of the trap’s form and boundary conditions on the BEC statistics in the interacting gas could be seen from the analysis of a mesoscopic system when the condensate boundary layers near the borders of the trap occupy only a small fraction of the trap’s volume in the Thomas-Fermi regime. The remaining, main part of the trap’s volume contains an almost homogeneous condensate and determines the main part of the structure and Bogoliubov couplings of all low energy excitations. The contributions from these excitations dominate the values of all higher-order generating cumulants ${\tilde{\kappa }}_{m},\;m\geq 3$, and, hence, are responsible for the non-Gaussian behavior of the BEC statistics. At the same time, the second generating cumulant, i.e., the variance, $\sigma^{2}∼{\tilde{\kappa }}_{2}$ could contain an appreciable contribution from the high energy, $\varepsilon_{j}∼\;\mathrm{or}\;>gn_{0}$, excitations the wavelengths of which are shorter than the healing length. The latter implies a validity of the WKB (quasi-classical) approximation for the solution to the Bogoliubov-de Gennes Equations (6) for these high energy quasiparticles even in the boundary layer of the healing-length thickness. It means that the high energy excitations propagate adiabatically (as in the geometrical optics approximation) through the boundary layer and, hence, fully feel the boundary conditions of the trap. As a result of this interplay between the different properties of the low and high energy excitations and their different roles in the BEC statistics, the scaled higher-order cumulants in Equation (26) and, hence, the statistics of condensate fluctuations in Equation (27) in the interacting gas depend on the trap’s geometry (form) and boundary conditions and are non-Gaussian. A very similar difference in the dependences of the second-order and higher-order cumulants exists also in the case of the varying interparticle interaction *g*, i.e., the parameter Δ in Equation (13), as is discussed in Section 5 and in Figure 1.

Of course, there are many effects which are not included in the formulated above basic model of the BEC statistics such as (a) the inhomogeneity of the condensate, in particular, within the boundary layers of the healing-length thickness near the borders of the trap, (b) the effect of such inhomogeneities on the modification of the quasiparticle energy spectrum, Bogoliubov couplings and squeezing of the noncondensate occupation, (c) the thermal and many-body effects in the range of temperatures from $T∼T_{c}/2$ to the critical temperature $T=T_{c}$ which are beyond the Bogoliubov approximation, etc. They require much more involved theoretical analysis and can considerably modify the BEC statistics in the interacting gas. Moreover, in order to describe correctly the full range of parameters and, in particular, an evolution of the BEC statistics in its transition from a small mesoscopic system to a macroscopic system or from an ideal gas with zero interaction g=0 to an interacting gas in the Thomas-Fermi regime as well as in order to get numerically accurate parameters of the BEC statistics one has to improve the basic model by the accurate account and solutions for these effects.

So, the intriguing problems of whether or not the nature allows the surface’s geometry (form) and boundary conditions of a macroscopically large trap to influence the BEC statistics and how the BEC statistics evolves with increasing trap’s size and interparticle interaction remain open. A similarly general question is whether or not the BEC statistics in the real macroscopic systems remains non-Gaussian in the thermodynamic limit. A universal mechanism leading to the Gaussian statistics in the many-body thermodynamic systems is well known. It is the central limit theorem valid for the sum of a large system of independent random variables in which there are no strongly dominating small subsystems. The considered systems significantly differ from the usual thermodynamic systems since the spontaneous symmetry breaking creates a well-developed condensate, or a coherent macroscopic wave function, which spreads over the entire volume of the system from border to border and coherently transfers the information about the trap’s form (geometry) and boundary conditions over the whole system. This could be the basis of the general mechanism keeping the BEC statistics non-Gaussian and dependent on the trap’s form and boundary conditions in the thermodynamic limit. Independently on the answers to these questions, the basic model presented in this paper can be employed as a useful constructive basis for the search, comparison and classification of various mechanisms affecting the BEC statistics.

Certainly, the ultimate answers to all these questions should be given by the experimental studies of the BEC statistics in the interacting gas. They constitute a promising field of research but require very accurate measurements and a very stable confinement of BEC with the precisely controllable parameters. A recent progress in the experimental techniques suggests that the BEC statistics is already becoming accessible experimentally (see, e.g., [47]). Among the recent experiments, especially promising are the experiments of the Hadzibabic’s group [48,49,50] with an almost flat cylindrical trap where the thermally-dominated regime seems to be accessible. For example, the setup of [49] in the thermally-dominated regime of condensate depletion at $T∼T_{c}/3$ constitutes a macroscopically large system since the parameter of the thermodynamic limit in Equation (13), $\alpha =\hbar^{2}\pi^{2}/\left(2ML^{2}T\right)$, is indeed very small, α∼0.003. Hence, the mesoscopic effects should be relatively weak and the described above anomalous thermodynamic-limit BEC statistics could be revealed experimentally. Another interesting example of the uniform BEC with the almost flat potential inside the box trap and vertical “walls” at the borders was demonstrated in the experiments of the Raizen’s group [51,52,53]. Other promising experimental setups include a two-dimensional flat trap of Dalibard’s group [54], the circular waveguides for BEC trapping [55] and the system which “paints” an arbitrary border of the trap by rapidly moving laser beams [56].

## 7. Conclusions

We formulated and solved the basic model (10)–(12) of the BEC statistics in a weakly interacting gas confined in a flat trap within the Thomas-Fermi approximation of the mean-field theory of Gross-Pitaevskii and Bogoliubov-de Gennes. This model yields the non-Gaussian condensate occupation statistics which is different from also non-Gaussian ideal-gas BEC statistics (for the same trap) but shares with it a similar dependence on the trap’s form and boundary conditions. Such a dependence does not vanish with the increase of the interparticle interaction, the number of trapped particles and the volume of the system, that is in the thermodynamic limit.
